# Glycaemic and insulinaemic impact of oats soaked overnight in milk vs. cream of rice with and without sugar, nuts, and seeds: a randomized, controlled trial

**DOI:** 10.1038/s41430-018-0329-1

**Published:** 2018-10-08

**Authors:** Thomas M. S. Wolever, Peter J. H. Jones, Alexandra L. Jenkins, Rebecca C. Mollard, Haizhou Wang, Alie Johnston, Jodee Johnson, YiFang Chu

**Affiliations:** 1 Glycemic Index Laboratories, Inc., Toronto, ON Canada; 2 Richardson Centre for Functional Foods and Nutraceuticals, University of Manitoba, Winnipeg, MB Canada; 3 Quaker Oats Center of Excellence, PepsiCo R&D Nutrition, Barrington, IL 60010 USA

**Keywords:** Homeostasis, Metabolic syndrome

## Abstract

****Background/Objectives**:**

Soaking oats overnight in milk renders them ready to eat the next morning, however, it is unknown whether oats prepared this way will retain its relatively low glycaemic and insulinaemic impact. Therefore, we compared the glycaemic, insulinaemic and subjective hunger responses elicited by oats soaked overnight in 110 g skim-milk (ONO) vs. cooked cream of rice cereal (CR), both with and without inclusions.

**Subjects/Methods:**

The project was performed at two research centers (Toronto, Winnipeg) as two separate studies each using a randomized, cross-over design with similar methods. The glycaemic and insulinaemic responses of overnight-fasted participants without diabetes (males:females: Toronto, 24:16; Winnipeg, 20:20) were measured for 3 h after consuming CR and ONO fed alone (Toronto) or with added sugar, nuts, and seeds (CRsns and ONOsns) (Winnipeg). Participants rated subjective hunger using visual analog scales. Data were analyzed by paired *t*-test. The primary endpoint was 0–2 h incremental area under the curve (iAUC) for glucose.

**Results:**

Mean glucose iAUC was 33% less, after ONO than CR (mean difference was 39 (51–27) mmol × min/l, *p* < 0.0001) and 24% less, after ONOsns than CRsns (mean difference was 43 (65–21) mmol × min/l, *p* = 0.0003). Serum-insulin iAUC was 33% less, after ONO than CR (mean difference 57 (81–40) pmol × hl, *p* < 0.0001) and 32% less, after ONOsns than CRsns (966 (1360–572) pmol × h/l, *p* < 0.0001). In both Toronto and Winnipeg, subjective hunger ratings were similar across the two treatments.

**Conclusions:**

Oats prepared by soaking overnight in skimmed milk without and with inclusions retain their relatively low glycaemic and insulinaemic impact.

## Introduction

Oats elicit a lower glycaemic response than most other breakfast cereals when comparing equivalent amounts of available carbohydrate (avCHO) [1–4]. Oats are rich in β-glucan, a highly viscous soluble dietary fiber [5] which, when added to test-meals, reduces postprandial blood-glucose, and insulin responses [6, 7] by increasing the viscosity of the contents of the upper gut [6, 7] and thereby delaying the absorption of carbohydrates [8]. Traditionally, oats are cooked in water prior to consumption, a process that not only gelatinizes starch causing more rapid digestion and an increased glycaemic response [9], but also solubilizes β-glucan which increases its ability to reduce the glycaemic response [10]. Another way to prepare oats is to soak them overnight in milk and consume them cold, the next morning (overnight oats). This reduces morning preparation time, and, thus, may encourage increased consumption of oats. However, it is unknown whether oats soaked overnight in skim-milk (ONO) will retain their relatively low glycaemic and insulinaemic impact.

There are evidence that the hot oatmeal elicits greater satiety [11] and a lower food intake [12] than Honey Nut Cheerios, a ready-to-eat cereal made from oats. However, it is unknown if ONO will promote greater satiety than a control cereal. In the present study we used cooked cream of rice cereal (CR) as a control because, while CR is similar to ONO in that neither are ready-to-eat cereals and both are consumed in a hydrated form, CR does not contain β-glucan, the hypothesized major active ingredient in oats. Furthermore, we previously compared the glycaemic impact of cooked oats to cooked CR [13, 14] so that, using CR as an anchor, the results for ONO can be compared to the previous results for hot oatmeal.

It has been suggested that comparing the glycaemic responses of individual foods has limited clinical utility because the difference in response will not be maintained when the foods are incorporated into a mixed-meal containing fat, protein and carbohydrate from other foods. Oats may be consumed in a mixed-meal containing milk and additions such as sugar, nuts, and seeds. We hypothesized that ONO, with or without such inclusions, would elicit lower glycaemic and insulinaemic responses than CR, but that the relative difference in glycaemic response between CR and ONO with added sugar, nuts, and seeds would be less than that between the cereals alone.

Therefore, we aimed to compare the glucose, insulin and subjective appetite responses elicited by ONO with those elicited by CR served alone and with the addition of sugar, nuts and seeds (inclusions) added to both cereals. We determined the effect of these inclusions because they would be present in a commercially available product and may influence the glycemic response.

## Materials and methods

The project was carried out in two research centers as two separate studies each with an open-label, randomized, cross-over design using similar methods. CR and ONO fed alone were compared at GI Labs, Inc., Toronto, Ontario (GI Labs), while CR and ONO with inclusions (CRsns and ONOsns, respectively) were compared at the Richardson Center for Functional Foods and Nutraceuticals, University of Manitoba, Winnipeg, Manitoba (RCFFN). The studies were registered at www.clinicaltrials.gov as NCT03150251 and NCT03091946.

### Participants

Participants were males and non-pregnant females aged 18–75 years with BMI ≥ 20.0 and < 35.0 kg/m² and fasting serum-glucose < 7.0 mmol/l (or blood-glucose < 6.3 mmol/l). Methods of recruitment and details of inclusion/exclusion criteria are given in supplementary information. The GI Labs protocol was approved by IRB Services, Inc.; the RCFFN protocol and recruiting advertisements were approved by the University of Manitoba Research Ethics Board; all participants provided a written informed consent prior to starting the study.

### Study design and procedures

Eligible participants were studied on separate days over a period of 2–6 weeks. The interval between successive tests was not less than 48 h. On every test day, the participants came to their respective research center in the morning after a 10–12 h overnight fast (water was allowed). Participants were asked to maintain stable dietary and activity habits throughout the study and to refrain from drinking alcohol, and from unusual levels of food intake or physical activity for 24 h before each test. If any subject was unwell or had not complied with the experimental conditions, the test was rescheduled for another day.

On each test occasion subjects were weighed, provided two fasting blood samples and rated their hunger. Then, the subjects consumed a test-meal which they were asked to consume within 10 min. At the first bite a timer was started and 10 additional blood samples were taken at intervals over the next 3 h. Subjective hunger was assessed at intervals of over 3 h in Toronto or 4 h in Winnipeg. During the 3–4 h of the test, participants remained seated quietly. After the last hunger rating had been completed, participants were offered a snack and allowed to leave. Details of procedures are given in supplementary information.

### Test-meals

At GI Labs the ONO test-meal consisted of 40 g oats (Quaker Kettle Hearty Overnight Oats, Chicago, IL) stirred into 110 g skim milk, soaked overnight (15–17 h) at 4 °C, and consumed cold with 160 ml water; the CR test-meal consisted of 28.8 g cream of rice cereal (B&G Foods, Inc., Parsippany, NJ, USA) stirred into 160 g water, microwaved on high for 3 min, stirred, left to sit for 1 min and served with 110 g skim milk. At GI Labs all subjects also tested CR, which had been soaked overnight in milk, but these results are not presented here as this does not represent how consumers eat the product.

At RCFFN the ONOsns test-meal consisted of a sachet containing 40.5 g oats plus 28.5 g of inclusions (9.3 g sucrose, 8.1 g sliced almonds, 4.6 g toasted coconut, 3.5 g white quinoa flakes, 2.3 g whole flaxseed, 0.4 g sodium chloride and 0.3 g natural flavors) stirred into 110 g skim milk soaked overnight (15–17 h) in a refrigerator, and consumed cold with 160 ml water; the CRsns test-meal consisted of a sachet containing 28.8 g cream of rice cereal plus 28.5 g of inclusions (same as added to ONOsns) stirred into 160 g water, microwaved on high for 3 min, stirred, left to sit for 1 min and served with 110 g skim milk.

The composition of the test-meals is shown in Table 1. At both centers test-meals were matched for avCHO and served with a drink of one or two cups of coffee or tea or water with 30 ml milk (2% at GI labs and skim at RCFFN) and non-caloric sweetener if desired; the type and volume of drink selected on the first visit was consumed on the subsequent visit. A description of the randomization procedures is given in supplementary information [15].

**Table 1:** Composition of test meals

	Abbrev.	Weight (g)	Energy (kJ (kcal))	Protein (g)	Fat	Carbohydrate (g)		
					(g)	Total	Fiber	Avail.
Overnight oats	ONO	40	628 (150)	5	4	27	4	23
Cream of rice	CR	28.8	427 (102)	1.9	0	23	0	23
Overnight oats plus sugar, nuts, and seeds	ONOsns	69	1210 (289)	8.3	10.4	43.8	6.3	37.5
Cream of rice plus sugar, nuts, and seeds	CRsns	57.3	1005 (240)	4.8	7.5	39.4	2.5	36.9
Skim milk^a^	–	110	155 (37)	3.7	0	5.5	0	5.5

Avail., available carbohydrate (total carbohydrate minus dietary fiber, rounded to nearest 0.1 g). Nutrition information for cereals provided by PepsiCo

^a^Nutrition information for milk based on the Nutrition Facts Table assuming the density of skim milk to be 1.036 g/ml; thus 110 g milk = 106 ml.

### Biochemical analysis

At GI Labs, finger-prick whole blood-glucose was measured using a YSI model 2300 STAT analyzer (Yellow Springs, OH, USA) and serum-insulin was measured using the Human Insulin EIA Kit (Alpco Diagnostics, Salem, NH, USA, catalog # 80-INSHU-E10.1). At RCFFN, finger-prick glucose was measured using a glucometer (StatStrip Glucose, Nova Biomedical Waltham, MA, USA) calibrated to provide values of plasma glucose [16] and serum human insulin was measured by immunoassay (Meso Scale Discovery, Rockville, MD, USA). Details of biochemical methods are given in supplementary information.

### Calculations

Incremental areas under the blood-glucose and serum-insulin response curves (iAUC), ignoring area below fasting, were calculated using the trapezoid rule [17, 18]. Fasting glucose and insulin were taken to be the mean of the concentrations in the two fasting samples. For subjective hunger scores, net incremental area under the curve (netAUC), and subtracting area below the baseline, was calculated using the trapezoid rule [11, 12].

### Statistical analysis

The data from each study were analyzed separately. At both centers the primary endpoint was glucose iAUC from 0 to 2 h (iAUC0–2). Secondary endpoints are given in supplementary information. Paired *t*-tests were used to determine whether differences were statistically significant, with the criterion for significance being a two-tailed *P* < 0.05. Response curves were analysed using repeated-measures analysis of variance (RMANOVA) examining for the main effects of time and test-meal, and the time × test-meal interaction. After demonstration of significant heterogeneity, RMANOVA was used to determine the effects of test-meal at the specific time points. With *n* = 40 subjects, each study had 80% power to detect a difference of 16%, and 90% power to detect a difference of 19% in the primary endpoint of glucose iAUC0–2.

The results of the two studies were compared with each other using Fisher’s exact tests for categorical variables or unpaired *t*-test for continuous variables.

## Results

**Fig. 1:**
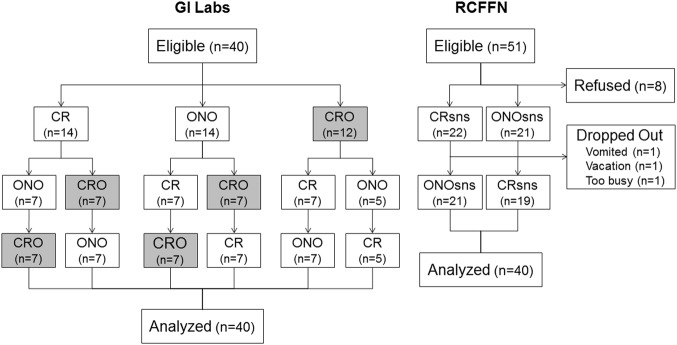
Flowchart. Footnote: GI Labs, Glycemic Index Laboratories, Inc., Toronto, Ontario tested Cream of Rice cereal (CR), oats soaked overnight in skim-milk (ONO) and cream of rice soaked overnight in skim-milk (CRO; results not presented here). RCFFN, Richardson Center for Functional Foods and Nutraceuticals, University of Manitoba, Winnipeg, Manitoba tested CR with added sugar, nuts and seeds (CRsns) and ONO with added sugar, nuts and seeds (ONOsns)

GI Labs recruited 40 eligible subjects, all of whom completed the study; RCFFN recruited 43 eligible subjects, 3 of whom did not complete the study leaving 40 who completed the study (Fig. 1). The characteristics of the participants who completed the studies are shown in Table 2.

**Table 2:** Subject characterisics

		GI Labs	RCFFN	*P*
Sex (M:F)		24:16	20:20	ns
Age (years)		39.2 ± 13.1	36.7 ± 16.2	ns
Weight (kg)		75.3 ± 11.9	72.8 ± 12.5	ns
Body mass index (kg/m²)		26.5 ± 3.1	24.8 ± 3.3	0.016
Fasting plasma glucose (mmol/l)		4.6 ± 0.4	5.2 ± 0.6	<0.0001
Blood pressure	systolic (mmHg)	120.1 ± 112.3	111.7 ± 14.0	0.006
	Diastolic (mmHg)	74.6 ± 9.9	72.0 ± 9.7	ns

Values are means ± SD

### Blood-glucose responses

**Fig. 2:**
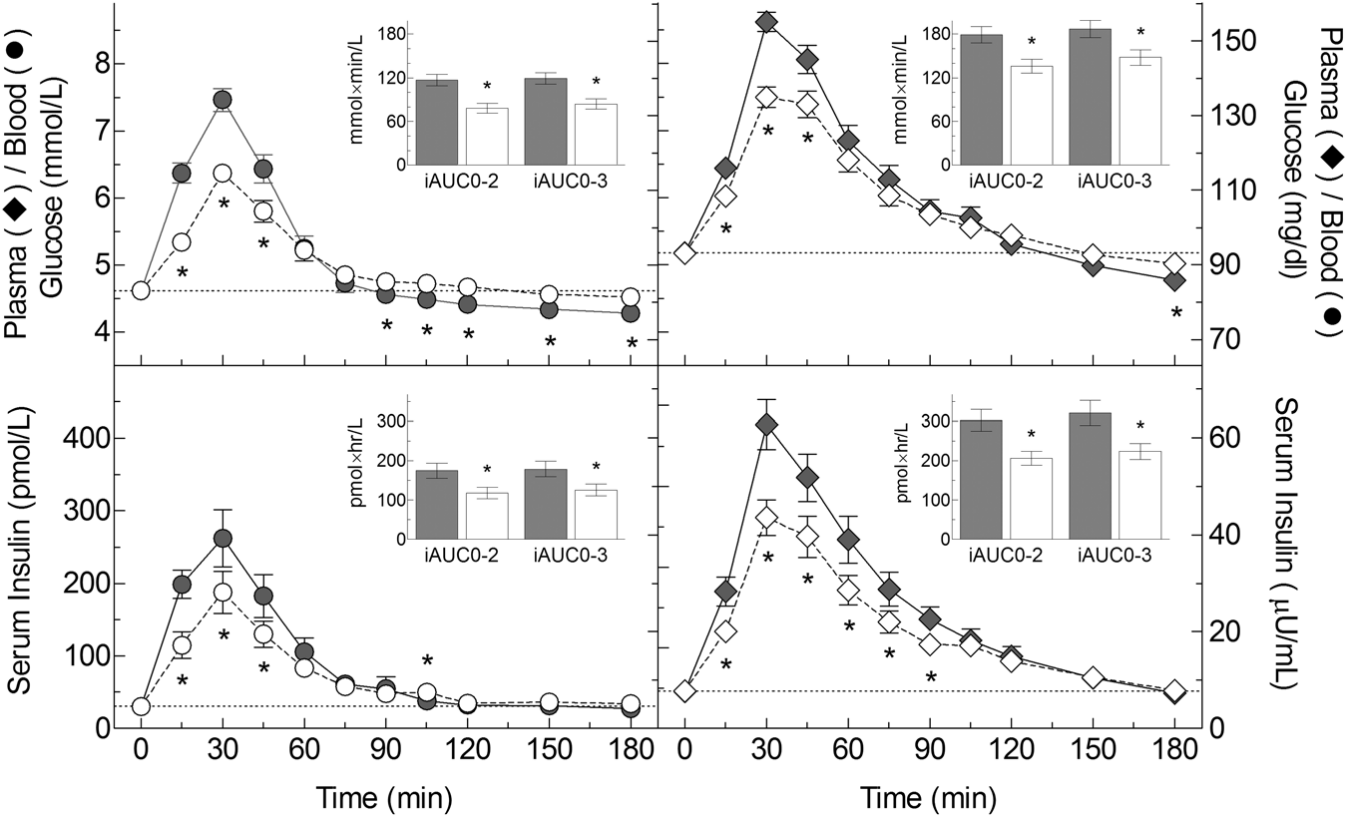
Glucose and insulin responses. Main graphs: blood or plasma glucose (top) and serum-insulin (bottom) concentrations elicited by cooked Cream of Rice cereal (filled symbols) and oatmeal soaked overnight in skim-milk (open symbols). Cereals were fed alone (circles) or contained added sugar, nuts, and seeds (diamonds). Points are means ± SEM for *n* = 40 subjects. Insets: Incremental areas under the curves of glucose and insulin from 0 to 2 h (iAUC0–2) and 0–3 h (iAUC0–3). Values are means ± SEM for *n* = 40 subjects. * Significant difference between cream of rice and overnight oats

Glucose response curves are shown in Fig. 2. Mean glucose iAUC from 0 to 2 h (iAUC0–2), after ONO and ONOsns were 33% (*P* < 0.0001) and 24% (*P* = 0.0003), less than after CR and CRsns, respectively (Table 3). Mean glucose iAUC0–2 after CRsns and ONOsns were significantly greater than the respective values after CR and ONO (*P* < 0.001 and *P* < 0.001). Similar effects within and between centers were seen for iAUC0–3 (*P* < 0.001 and *P* < 0.001) and peak rise (*P* < 0.001 and *P* < 0.001) (Table 3).

**Table 3:** Primary and major secondary endpoints for glucose and insulin

	GI Labs (*n* = 40)		RCFFN (*n* = 40)	
	CR	ONO	CRsns	ONOsns
*Glucose*				
iAUC0–2 (mmol × min/l)	117.2 ± 49.5	78.3 ± 41.8*	178.9 ± 71.4^†^	135.9 ± 60.4^†^*
iAUC2–3 (mmol × min/l)	1.7 ± 3.6	5.4 ± 7.4*	7.6 ± 9.7*	12.1 ± 15.3*
iAUC0–3 (mmol × min/l)	118.9 ± 49.6	83.7 ± 44.4*	186.5 ± 75.2^†^	148.0 ± 67.2^†^*
Peak rise (mmol/l)	2.97 ± 0.80	1.87 ± 0.60*	3.72 ± 0.89^†^	2.72 ± 0.87^†^*
Fasting (mmol/l)	4.62 ± 0.40	4.62 ± 0.42	5.18 ± 0.51	5.17 ± 0.48
2 h (mmol/l)	4.41 ± 0.45^‡^	4.67 ± 0.40*	5.31 ± 0.80	5.44 ± 0.54^‡^
2 h increment (mmol/l)	−0.21 ± 0.42	0.05 ± 0.36*	0.13 ± 0.68^†^	0.27 ± 0.65^‡^
Above:below fasting at 2 h	13:27	22:18^a^	22:18^b^	26:14
*Insulin*				
iAUC0–2 (pmol × h/l)	174.6 ± 120.7	117.6 ± 90.1*	302.3 ± 175.3^†^	205.7 ± 113.9^†^*
iAUC2–3 (pmol × h/l)	4.2 ± 7.3	8.1 ± 11.8	22.7 ± 32.8*	19.2 ± 21.6*
iAUC0–3 (pmol × h/l)	178.7 ± 123.0	125.7 ± 92.4*	325.0 ± 196.9^†^	225.9 ± 129.8^†^*
Peak rise (pmol/l)	278 ± 197	185 ± 146*	367 ± 180^†^*	250 ± 139^†^

Results are given as means ± SD for *n* = 40 subjects

*iAUC* incremental area under the curve

*Differs significantly from respective CR or CRsns by paired *t*-test (*p* < 0.05)

^†^Differs significantly from corresponding mean from GI Labs by unpaired *t*-test (*p* < 0.05)

^‡^Differs significantly from fasting glucose by paired *t*-test (*p* < 0.05)

^a^Percentage above fasting after ONO differs from CR by Fisher’s exact test (*p* < 0.05)

^b^Percentage above fasting after CRsns differs from CR by Fisher’s exact test (*p* < 0.05)

**Fig. 3:**
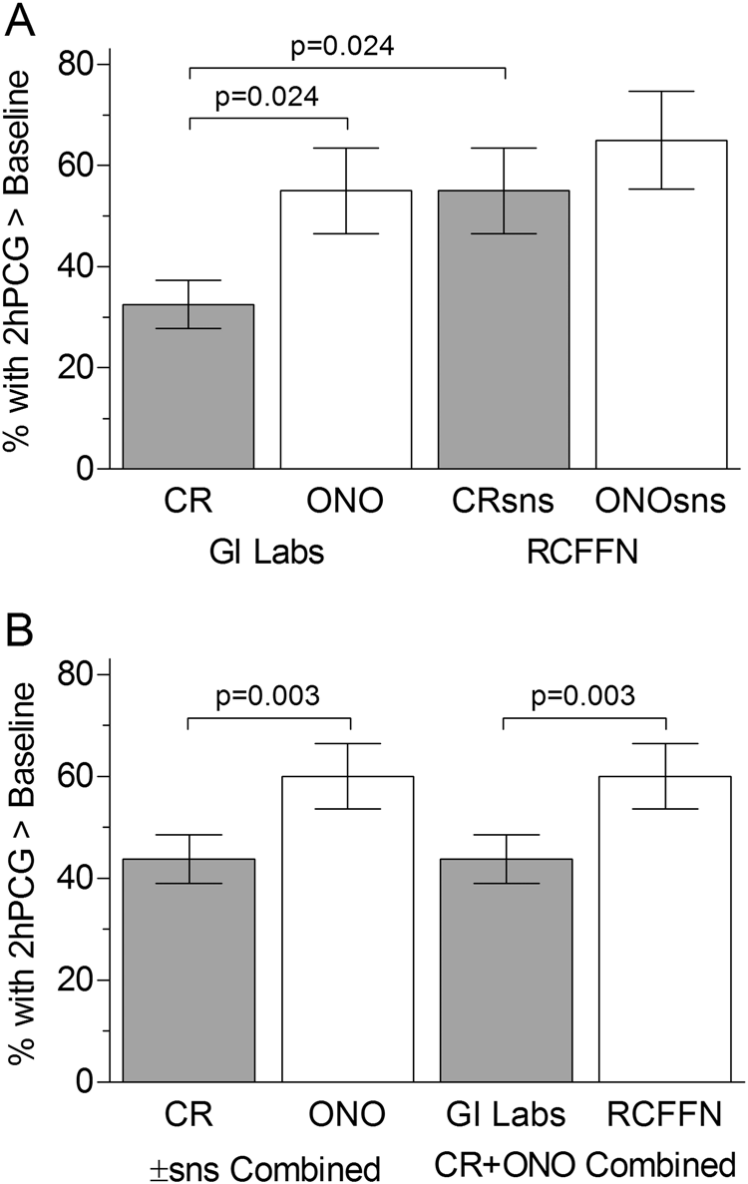
Glucose above baseline at 2 h. Percentage of subjects with glucose 2 h after eating (2hPCG) greater than baseline (fasting glucose). Bars are percentages ± 95% confidence intervals. **a** Data for individual test-meals (each bar represents the percentage of *n* = 40 subjects). **b** Each bar represents the percentage of *n* = 80 subjects; CR, CR and CRsns combined; ONO, ONO and ONOsns combined; GI Labs, CR and ONO combined; RCFFN, CRsns and ONOsns combined

Blood-glucose was significantly above baseline at 2 h after ONOsns (*P* = 0.013), similar to baseline at 2 h after ONO and CRsns, and below baseline at 2 h after CR (*P* = 0.003; Table 3). Significantly more subjects had 2 h glucose above baseline after ONO (55%) than CR (32.5%) (*P* = 0.024), but the difference between ONOsns (65%) and CRsns (55%) was not significant (Table 2; Fig. 3). More subjects had glucose above baseline at 2 h after CRsns compared to CR (55% vs. 32.5%, *P* = 0.023), but the difference between ONOsns (65%) and ONO (55%) was not significant.

### Serum-insulin responses

Insulin response curves are shown in Fig. 2. Mean insulin iAUC from 0 to 2 h (iAUC0–2), iAUC from 0 to 3 h (iAUC0–3) and peak rise after ONO and ONOsns were significantly higher than their respective values after CR and CRsns (Table 3). Mean insulin responses after CRsns and ONOsns were greater than the respective values after CR and ONO for iAUC0–2, iAUC2–3 and iAUC0–3 (Table 3).

### Subjective hunger

Subjective hunger ratings after CR and CRsns were similar to those after ONO and ONOsns (Supplementary Figure 1). Subjective ratings for desire to eat, fullness, and prospective consumption after ONO were similar to those after CR (not shown). Mean hunger rating was significantly below baseline at 2 h after both CR (mean difference (95% confidence-interval)), 10.3 (18.2–2.3) mm (*P* = 0.013) and ONO, 9.7 (18.1–1.4) mm (*P* = 0.023) but did not differ significantly from baseline at 2 h after CRsns and ONOsns.

## Discussion

ONO elicited a significantly lower glycaemic response than CR, regardless of whether the cereals were tested alone or with the addition of sugar, nuts and seeds. The glycaemic profiles after ONO and ONOsns were flatter than those after CR and CRsns with a lower peak rise, higher glucose at 120 or 180 min, and a larger percentage of subjects with glucose above baseline at 2 h.

The subjects at RCFFN who consumed the test-meals with added sugar, nuts, and seeds tended to be younger and had significantly lower BMI and systolic blood pressure than those at GI Labs; despite this, RCFFN subjects had significantly higher fasting glucose and significantly higher postprandial glucose and insulin responses. The higher fasting glucose in RCFFN subjects (Fig. 1) is explained by the fact that at RCFFN glucose was measured using a glucometer calibrated to give plasma glucose [19], which yields values approximately 0.7 mmol/l higher than whole blood-glucose, as measured by GI Labs [19]. The ~60% higher glucose iAUC in RCFFN subjects is partly explained by the higher avCHO content of the test-meals they consumed and partly because using a glucometer calibrated to give plasma glucose yields a significantly higher iAUC than measuring whole blood-glucose [19]. Nevertheless, the method of measuring glucose would not be expected to have a significant effect on the glycaemic response after ONO relative to CR [19].

The clinical relevance of studies comparing the glycaemic responses of individual foods has been questioned because responses will differ when the foods are consumed as part of mixed meals containing other sources of carbohydrate, fat and/or protein [20–23]. However, we believe it is clinically appropriate to compare glycaemic responses of individual foods because the effect on the glycaemic response of adding other sources of carbohydrate, fat and/or protein to the individual food can be estimated from data on the dose-response effects of various types of carbohydrate, fat and protein on glycaemic responses [17, 24–27]. For example, here we determined the glycaemic responses elicited by ONO and CR both with and without inclusions. Relative to CR, the mean glycaemic response after ONO (no inclusions), 67%, tended to be less than that for ONOsns (with inclusions), 76%. However, this difference is almost exactly that expected based on the composition of the inclusions. In Supplementary Information, we show how the carbohydrate, protein and fat content of the inclusions predict that ONOsns would elicit a glycaemic response 77% of CRsns. It is not feasible to test every one of the virtually infinite number of possible meals containing ONO; our results suggest this is not necessary since the relative glycaemic impact of mixed meals can be determined from their component parts.

Mean insulin iAUC0–2 h, iAUC0–3 and peak rise were significantly lower after ONO than CR and significantly lower after ONOsns than CRsns. The % reductions in insulin were similar to or greater than those for glucose. This is important because it has been suggested that reductions in glycaemic response are only beneficial if they are accompanied by reductions in insulin response of a similar (or greater) magnitude [28, 29]. Reduced postprandial glucose responses can be due either to a reduced rate of entry of glucose into the circulation [30] or to reduced hepatic glucose production [31] or to an increased rate of glucose disposal because of an increased insulin response. Since we found no evidence of disproportionately high insulin responses, the present results support the hypothesis that ONO and ONOsns reduced glycaemic responses because they contain slowly absorbed carbohydrates.

A marker of slow carbohydrate absorption could be glucose above fasting at 2 h after eating, and we found that the percentage of subjects with glucose above baseline at 2 h was significantly higher after ONO than CR. However, whether an individual’s blood-glucose is above fasting at 2 h depends not only on whether dietary carbohydrates are slowly absorbed, but also on how much carbohydrate is consumed, the individual’s carbohydrate tolerance (carbohydrate intolerance is defined by high blood-glucose 2 h after eating), and perhaps other factors. The fact that RCFFN subjects consumed a larger test-meal may explain why, overall, more subjects at RCFFN had glucose above baseline at 2 h than at GI Labs.

Subjects at RCFFN had lower subjective hunger ratings than those at GI Labs, which could have been due to a shorter fast or an earlier start time, or to differences in subject perceptions. The subjective nature of hunger ratings is also shown by the counter-intuitive finding that subjects at GI Labs reported being less hungry than baseline 2 h after consuming 580–780 kJ (140–90 kcal) test-meals, whereas subjects at RCFFN reported being as hungry as at baseline 2 h after consuming 1160–1370 kJ (280–330 kcal) test-meals. We were unable to demonstrate any significant difference in subjective hunger ratings after ONO compared to CR or ONOsns compared to CRsns.

We did not compare the glycaemic response elicited by ONO with that of cooked oatmeal. However, we previously compared the glycaemic impact of various types and amounts of cooked oats to those of avCHO-matched portions of cooked CR [13, 14]. Thus, by using CR as an anchor, the results for ONO in this study can be compared with those of hot oatmeal in the previous studies. Here, ONO elicited an iAUC0–2h (mean ± SEM) 69.1 ± 4.6% of that elicited by CR, a value significantly less than that for 40 g Quick Oatmeal (Quaker, Cedar Rapids, IA), 89.9 ± 7.2% (*P* = 0.018) and significantly less than the mean for the 30, 40, and 60 g servings of oatmeal, 81.9 ± 3.3% (*P* = 0.029) despite the fact that the Quick Oatmeal contained 40% more dietary fiber per 40 g serving than ONO. The relative response for ONO, 69.1 ± 4.6%, was also significantly less than those for Quaker Instant Oatmeal (104.4 ± 5.1%, *P* < 0.05) and Quaker old fashioned oats (92.4 ± 6.7%, *P* < 0.05) and similar to that for Quaker steel cut oatmeal (72.4 ± 6.3), each tested in *n* = 30 subjects [14]. Taken together, these results suggest that soaking oats overnight in milk may reduce their glycaemic impact relative to hot oatmeal, however, this must be confirmed by a direct comparison of cooking vs. soaking.

The strengths of the study include the relatively large number of subjects tested in each of two different centers using the same blood sampling schedule and the same test-meals, except for the addition of sugar, nuts, and seeds at RCFFN, inclusions that would likely be found in commercial ONO products. The fact that the centers used different analytical methods for glucose and insulin and that the two population studied differed in several respects serve to enhance the robustness and applicability of the findings.

It is concluded that oats soaked overnight in skim-milk elicit significantly lower glycaemic and insulinaemic responses than cooked Cream of Rice cereal whether the cereals are consumed alone or with inclusions. The findings that significantly more participants had glucose above fasting 2 h after consuming ONO than 2 h after CR, with or without inclusions, support the hypothesis that overnight oats contain slowly absorbed carbohydrate.

## Electronic supplementary material

Supplemental Material
