# Supplementary material for: Glycaemic and insulinaemic impact of oats soaked overnight in milk vs. cream of rice with and without sugar, nuts, and seeds: a randomized, controlled trial
Source: Eur J Clin Nutr. 2018 Oct 8;73(1):86–93. doi: 10.1038/s41430-018-0329-1 (PMC6326951; doi:10.1038/s41430-018-0329-1)
Supplement: Supplementary file 1 — Supplemental Material [file 41430_2018_329_MOESM1_ESM.docx]

**Subject Recruitment and Consent**

At GI Labs participants were recruited from the pool of volunteers who had previously participated in studies at GI Labs and had given permission to be contacted for future studies; at RCFFN, participants were recruited from posters, e-newsletters, site-affiliated websites and database of participants who had given permission to be contacted for future studies.

Participants willing to be considered were invited to come to their respective research centre to have the study procedures explained to them and be given a copy of the consent form which they could either sign then, take away to sign at a later date, or decline to participate. Participants were encouraged to ask any questions they may have had and not to sign the consent form until all of their questions had been answered to their satisfaction. Those who consented to participate attended a pre-selection visit at which time subject eligibility was determined by asking questions about medical history and drug use, measuring their height and weight and calculating the BMI. If a fasting glucose measured within the last 3 months was not available, arrangements were made to have it measured.

**Details of Procedures**

On each test occasion the following occurred: at GI Labs, subjects were weighed and 2 fasting blood samples were obtained by finger-prick 5 min apart; at RCFFN a fasting finger-prick sample for glucose was obtained (-5 min), then subjects were weighed, had an indwelling catheter inserted into a forearm vein from which blood was drawn for insulin and a second finger-prick blood sample for glucose obtained (0 min). At both centers, after the 2^nd^ fasting finger-prick blood sample, subjects started to consume a test meal which was consumed within 10 min. At the first bite a timer was started and additional blood samples were taken at 15, 30, 45, 60, 75, 90, 105, 120, 150 and 180 min after starting to eat. At GI Labs, each blood sample consisted of 2-3 drops into a fluoro-oxalate tube for glucose analysis and 6-8 drops into a separate 0.3 ml Microvette (Sarsted Inc., Numbrecht, Germany) for insulin analysis. At RCFFN each blood samples at each time point consisted of a drop of finger-prick blood for glucose analysis and venous blood for insulin collected into 3.5 ml BD Gold SST tubes (Becton & Dickinson, Mississauga, ON). If a participant’s hands were cool, hands were warmed with an electric heating pad for 3-5 min prior to each sample.

At GI Labs, subjective desire to eat, hunger, fullness and prospective consumption were assessed at -5, 0, 15, 30, 45, 60, 75, 90, 105, 120, 150 and 180 min (after each blood sample had been obtained) using the Motivation to Eat questionnaire (15). At RCFFN subjective hunger was assessed at the same times listed above and also at 210 and 240 min. Subjective hunger was assessed using a visual analog scale (VAS) consisting of a 100 mm horizontal line anchored at the left end with “not hungry at all” and at the right end with “as hungry as I have ever felt”; subjects made a vertical mark along the line to indicate their feelings at that moment. Similar VAS were used to assess desire to eat, fullness and prospective consumption.

**Details of Biochemical analysis**

At GI Labs, immediately after collection, the tubes containing blood for glucose analysis were rotated to mix the blood with the anti-coagulant and then placed in a refrigerator until the last blood sample in the set had been collected; after 3 h the set of 12 tubes were bundled together with a rubber band and stored at -20˚C until analysis of whole blood glucose which was performed within 3 days using a YSI model 2300 STAT analyzer (Yellow Springs, OH). The microvette tubes containing blood for insulin were left at room temperature to allow the blood to clot, centrifuged and the serum transferred to labeled polypropylene tubes and stored at ‑70˚C prior to analysis of insulin using the Human Insulin EIA Kit (Alpco Diagnostics, catalog # 80-INSHU-E10.1). The lower limit of detection for this assay is 3.0 μU/mL; for statistical analysis undetectable values were assigned a value of 2.99 μU/mL.

At RCFFN, finger-prick glucose was measured using a glucometer (StatStrip Glucose, Nova Biomedical Waltham, MA) calibrated to provide values for plasma glucose (16). allowed to clot at room temperature for 30 min, centrifuged and the serum transferred to labeled micro-tubes and stored at -80ºC prior to analysis of human insulin analysis by immunoassay (Meso Scale Discovery, Rockville, MD).

**Secondary Endpoints**

At GI Labs the secondary endpoints were glucose iAUC2-3 and iAUC0-3, glucose increment at 2 h, insulin iAUC0-2, iAUC2-3 and iAUC0-3, peak concentrations and peak rises for glucose and insulin, glucose and insulin concentrations and increments at each time point over 3 h, hunger netAUC0-2, netAUC2-3 and netAUC0-3 and hunger increment at 2 h. At RCFFN the secondary endpoints were hunger increments at 3 and 4 h.

**Randomization**

At GI Labs the order of treatments was randomly assigned using the RAND() function (Excel, Microsoft Office 2010, Microsoft Canada, Inc., Mississauga, ON) to generate 48 orders which were assigned to subjects in the order they attended for the first visit with 8 extra orders to replace drop-outs if necessary. At RCFFN, the two ways in which two treatments could be ordered were randomly ordered 24 times for males and 24 times for females to yield 48 orders. Treatment allocation orders were sealed in sequentially numbered opaque envelopes by a third party and assigned to participants before their first visit. The additional 8 orders were included, if necessary, to assign to participants who replaced drop-outs.

**Expected Effect on Relative Glycemic Response of Adding Inclusions to the Test Meals**

To estimate the effect of the inclusions (sugar, nuts and seeds) to the CR and ONO test meals, 3 separate calculations are involved; first the mixed meal glycemic index (mmGI) must be calculated for each test meal, then the effect of the available carbohydrate (avCHO) content on the inclusions must be accounted for, and finally adjustment made for the fat and protein content of the inclusions.

The mmGI is calculated from the glycemic index (GI) values of the meal components and their content of avCHO. The GI of milk was taken to be 32. The GI of CR is not known, but was estimated from the mean GI values for puffed rice cereals (n=6, mean 85.8), puffed wheat cereals (n=2, mean 73.5) and cooked cream of wheat (n=2, mean 70) taken from ([www.glycemicindex.com](http://www.glycemicindex.com)) by assuming that: (GI puffed wheat)/(GI cream of wheat) = (GI puffed rice)/(GI cream of rice); thus the GI of CR = 85.8×70/73.5 = 81.5 (rounded to 82). The mmGI is the sum of GI×%avCHO/100 for each food in the test meal and is shown for CR and ONO in Supplementary Table 2. The mmGI for the CR test-meal was 72.4. The GI of ONO was estimated from mmGI of the CR test meal, and the fact that, since the ONO and CR test meals contained equivalent amounts of carbohydrate, the mean glycemic response (GR, iAUC0-2h) of ONO relative to CR (78.3/117.2 = 0.668) is equivalent to the calculated mmGI of the ONO test-meal relative to the CR test-meal: i.e. (mmGI of ONO test meal) / 72.4 = 0.668; thus, mmGI = 72.4×0.668 = 48.3. Since ONO and milk (GI=32), respectively contributed 80.7 and 19.3% of the avCHO in the ONO test-meal, (GI ONO)×0.807 + 32×0.193 = 48.3, the GI of ONO = (48.3-6.2)/0.807 = 52 (Supplementary Table 2).

The estimation of the mmGI of the inclusions is shown in Supplementary Table 3, and the calculation of the mmGI for the ONOsns and CRsns test meals in Supplementary Table 4.

It has been suggested^[[1]](#footnote-1)^ that the glycemic response (iAUC0-2h) elicited by test meals containing various amounts of different carbohydrates (assuming equivalent intakes of protein and fat), expressed relative to that elicited by 50g glucose, is given by the following equation:

RGR = 1.49×GI×(1-e^-0.0222g^) [1]

where GI is glycemic index of the food or mmGI of the test-meal and g is the amount of avCHO in the food or test-meal consumed. Thus, RGR adjusts for differences in avCHO content and GI of the test-meals. Adding protein and fat to carbohydrate results in dose-dependent reductions in glycemic response. For protein (% reduction per gram protein per 50g avCHO) the effect varies from ~0.6% for tuna, to ~1.4% for whey^[[2]](#footnote-2)^; for fat for butter or margarine the effect is similar at about 0.5% per gram fat per 50g avCHO.

Given that, at GI Labs, ONO elicited a mean glycemic response 66% of that for CR, then these calculations predict that ONOsns will elicit a mean glycemic response 77% of that for CRsns at RCFFN; a prediction which is virtually identical to the observed difference of 76%. Sensitivity analysis indicates that result of the prediction is robust, since changing the GI of CR or the inclusions by ±10 only changes the predicted relative response by ~±1% and changing the predicted impact of fat and protein has virtually no effect since the same amounts of fat and protein were added to both ONO and CR. The predicted RGR values for ONO and CR with inclusions are higher than those for ONO and CR without inclusions because of the former contain more avCHO than the latter; however, this difference does not predict the observed difference between the test meals with and without inclusions because different subjects were studied in the 2 centres; predicted RGR and adjRGR will only predict the relative glycemic impact of test-meals consumed by the same group of individuals.

**Supplementary Table 1: Inclusion / Exclusion Criteria**

*Inclusion Criteria:*

- Male or non-pregnant females, 18-75 years of age, inclusive

- Body mass index (BMI) ≥ 20.0 and < 35 kg/m² at screening (visit 1).

- Willing to maintain habitual diet, physical activity pattern, and body weight throughout the trial and to refrain from smoking for 12hr prior to each visit.

- Willing to maintain current dietary supplement use throughout the trial. On test days, subject agrees not to take any dietary supplements until dismissal from GI labs. Failure to comply will result in a rescheduled test visit.

- Normal fasting serum glucose (<7.0mmol/L capillary corresponding to whole blood glucose <6.3mmol/L).

- Willing to abstain from alcohol consumption and avoid vigorous physical activity for 24 h prior to all test visits.

- Absence of health conditions that would prevent fulfillment of study requirements as judged by the Investigator on the basis of medical history.

- Understanding the study procedures and willing to provide informed consent to participate in the study and authorization to release relevant protected health information to the study investigator.

*Exclusion criteria:*

- Failure to meet any one of the inclusion criteria

- Known history of AIDS, hepatitis, a history or presence of clinically important endocrine (including Type 1 or Type 2 diabetes mellitus), cardiovascular (including, but not limited to, atherosclerotic disease, history of myocardial infarction, peripheral arterial disease, stroke), pulmonary, biliary or GI disorders.

- Use of medications known to influence carbohydrate metabolism, including, but not limited to adrenergic blockers, diuretics, thiazolidinediones, metformin and systemic corticosteroids within 4 weeks of the screening visit, or with any condition which might, in the opinion of Dr. Wolever, the president of GI Testing, either: 1) make participation dangerous to the subject or to others, or 2) affect the results.

- Major trauma or surgical event within 3 months of screening.

- Unwillingness or inability to comply with the experimental procedures and to follow GI Labs safety guidelines.

- Known intolerance, sensitivity or allergy to any ingredients in the study products.

- Extreme dietary habits, as judged by the Investigator (i.e. Atkins diet, very high protein diets, etc.).

- Uncontrolled hypertension (systolic blood pressure ≥160 mm Hg or diastolic blood pressure ≥100 mm Hg as defined by the average blood pressure measured at screening.

- Change in body weight of >3.5kg within 4 weeks of the screening visit.

- Presence of any signs or symptoms of an active infection within 5 d prior to any test visit. If an infection occurs during the study period, test visits should be rescheduled until all signs and symptoms have resolved and any treatment (i.e. antibiotic therapy) has been completed at least 5 d prior to each test visit.

- History of cancer in the prior two years, except for non-melanoma skin cancer.

- Recent history (within 12 months of screening) or strong potential for alcohol or substance abuse. Alcohol abuse is defined as > 14 drinks per week (1 drink=12 oz beer, 5 oz wine, or 1.5 oz distilled spirits).

- Exposure to any non-registered drug product within 30 d prior to screening.

**Supplementary Table 2: Calculation of Mixed Meal GI (mmGI) for CR and ONO**

| Cream of Rice Test Meal | | | | | Overnight Oats Test Meal | | | | |
| --- | --- | --- | --- | --- | --- | --- | --- | --- | --- |
| Food | GI | avCHO | avCHO% | mmGI | Food | GI | avCHO | avCHO% | mmGI |
| CR | 82 | 23.0 | 80.7 | 66.2 | ONO | 52 | 23.0 | 80.7 | 42.2 |
| Milk | 32 | 5.5 | 19.3 | 6.2 | Milk | 32 | 5.5 | 19.3 | 6.2 |
| Mixed Meal GI | | | | 72.4 | Mixed Meal GI | | | | 48.3 |

**Supplementary Table 3: GI of Inclusions**

|  | g | avCHO (g/100g)* | avCHO (g) | avCHO  (% of total) | GI** | mmGI |
| --- | --- | --- | --- | --- | --- | --- |
| White Quinoa Flakes | 3.5 | 57.2 | 2.00 | 15.1 | 53 | 8.0 |
| Whole Flaxseed | 2.3 | 2.7 | 0.06 | 0.5 | 70 | 0.3 |
| Sugar | 9.3 | 100 | 9.30 | 70.3 | 62 | 43.6 |
| Toasted Coconut | 4.6 | 18.2 | 0.84 | 6.3 | 54 | 3.4 |
| Almond slices | 8.1 | 12.7 | 1.03 | 7.8 | 30 | 2.3 |
| Mixed Meal GI | | | | | | 57.7 |

* taken from USDA nutrient database; avCHO = carbohydrate – dietary fiber.

** taken from [www.glycemicindex.com](http://www.glycemicindex.com) (coconut = coconut sugar; almonds estimated).

**Supplementary Table 4: Calculation of Mixed Meal GI (mmGI) for CRsns and ONOsns**

| Cream of Rice plus Inclusions Test Meal | | | | | Overnight Oats plus Inclusions Test Meal | | | | |
| --- | --- | --- | --- | --- | --- | --- | --- | --- | --- |
| Food | GI | avCHO | avCHO% | mmGI | Food | GI | avCHO | avCHO% | mmGI |
| CR | 82 | 23.0 | 54.2 | 44.5 | ONO | 52 | 23.3 | 54.2 | 28.3 |
| Inclusions | 58 | 13.9 | 32.8 | 19.0 | Inclusons | 58 | 14.2 | 33.1 | 19.2 |
| Milk | 32 | 5.5 | 13.0 | 4.2 | Milk | 32 | 5.5 | 12.8 | 4.1 |
| Mixed Meal GI | | | | 67.6 | Mixed Meal GI | | | | 51.6 |

**Supplementary Table 5: Estimated Effect of Inclusions on Glycemic Responses.**

|  | mmGI | avCHO | RGR* | Protein** | | | Fat** | | | adjRGR^†^ | ONO as %CR |
| --- | --- | --- | --- | --- | --- | --- | --- | --- | --- | --- | --- |
|  |  |  |  | g | diff | adj | g | diff | adj |  |  |
| CR | 72.4 | 28.5 | 50.6 | 5.6 | 0 | 1 | 0 | 0 | 1 | 50.6 |  |
| ONO | 48.3 | 28.5 | 33.7 | 8.7 | 0 | 1 | 4 | 0 | 1 | 33.7 | 0.67 |
| CRsns | 67.6 | 42.4 | 61.4 | 8.5 | 2.9 | 0.96 | 7.5 | 7.5 | 0.96 | 56.3 |  |
| ONOsns | 51.6 | 43 | 47.3 | 12 | 3.3 | 0.95 | 10.4 | 6.4 | 0.96 | 43.4 | 0.77 |

*Calculated from equation [1] in supplementary text above.

** Difference in protein and fat between means with and without inclusions. Each gram of additional protein per 50g avCHO reduces RGR by 1.2% and each gram of additional fat per 50g avCHO by 0.5%.

† adjRGR = RGR × protein adj × fat adj.

**Supplementary Figure 1: Subjective Hunger.**

Hunger ratings elicited by cooked Cream of Rice cereal (filled symbols) and oatmeal soaked overnight in skim milk (open symbols). Cereals were fed alone (circles) or contained added sugar, nuts and seeds (diamonds). Points are means±SEM for n=40 subjects.

1. Wolever TMS. *The Glycaemic Index: A Physiological Classification of Dietary Carbohydrate*. CABI Publishing, Wallingford, UK, 2006, page 66. [↑](#footnote-ref-1)
2. Wolever TMS. Effect of macronutrients on the glycemic index. Am J Clin Nutr 2017;106:704-5. [↑](#footnote-ref-2)
